# Predicting Chikungunya disease personal protective behaviors: Results of a cross-sectional survey of US-Caribbean travelers

**DOI:** 10.15171/hpp.2020.08

**Published:** 2020-01-28

**Authors:** Kristina R Anderson, Kevin Naaman, Edna Omodior, Grace Karikari, Lori Pennington-Gray, Oghenekaro Omodior

**Affiliations:** ^1^Department of Recreation, Park, and Tourism Studies, School of Public Health, Indiana University, Bloomington, USA; ^2^Department of Counseling and Educational Psychology, School of Education, Indiana University, Bloomington, USA; ^3^Department of Applied Health Sciences, School of Public Health, Indiana University, Bloomington, USA; ^4^Department of Tourism, Recreation and Event Management, University of Florida, Gainesville, USA

**Keywords:** Caribbean region, Chikungunya virus, Health education, Protective factors, Travel-related illness

## Abstract

**Background:** Incidents of vector-borne disease have recently tripled in the United States. Chikungunya disease is a particularly common disease in the Caribbean, posing a threat to international tourists. However, the relationship between psychological variables derived from the protection motivation theory (PMT), and adoption of protective behaviors against the disease, is uncertain. This study sought to identify the psychological predictors of travelers’ protective health behaviors, specifically (1) appropriate clothing use, and (2) indoor spatial repellent use.

**Methods:** An online, retrospective survey of U.S. international travelers to Caribbean destinations measured the five constructs of the PMT in the context of Chikungunya disease: Perceived severity, perceived vulnerability, perceived response efficacy, perceived self-efficacy, and knowledge. Hierarchical logistic regression analyzed whether these five theoretical constructs predicted the two protective behaviors in respondents who met study criteria (n = 184).

**Results:** Results suggest that the interaction between chikungunya knowledge and perceived chikungunya severity predicts both appropriate clothing use (odds ratio [OR]: 1.95, CI: 1.18-3.25, *P* =0.010) and indoor spatial repellent use (OR: 1.55, CI: 1.05-2.29, *P* =0.029). In the cases of appropriate clothing use, the interaction between perceived chikungunya severity and perceived vulnerability was also a significant predictor (OR: 9.67, CI:1.23-75.80, *P* =0.031). Additionally, indoor spatial repellent use was also predicted by the interaction of chikungunya knowledge and perceived vulnerability (OR: 1.88, CI:1.18-3.02, *P* =0.009).

**Conclusion** : Two-pronged educational approaches may be most efficacious in increasing protective health behaviors. Such efforts could reduce incidents of chikungunya disease and other vectorborne diseases in travel destinations featuring high exposure risks.

## Introduction

In the United States, incidents of vector-borne diseases tripled from 2004-2016.^1^ Dengue, Zika, and Chikungunya as viral diseases transmitted by mosquitoes have spread to new regions, and concerns about further spread have become particularly salient in light of increasing international travel and changing climatic conditions.^2,3^ From 2013 through 2017, fewer than 200 cases of Chikungunya disease were reported in the United States, with the exception of 2014, when more than 2800 cases were reported.^4^ While this might suggest a 2014 peak and subsequent decline, recent ecological niche and climate modelling work suggests geographic expansion of*Aedes aegypti—* the mosquito transmitting the Chikungunya virus*—* into areas worldwide that have not experienced outbreaks previously.^5^ While Chikungunya rarely results in death, its symptoms can be severe and disabling; common symptoms include fever and severe joint pain.^6^ In 2018, the U.S. Food and Drug Administration added Chikungunya disease to its priority list for drug development; currently, there is neither a vaccine to prevent Chikungunya disease nor antiviral treatments.^6,7^ As a result, adoption of personal protection behaviors such as 1) use of insect repellents, 2) wearing long-sleeve shirts and pants, and 3) staying indoors or in screened-in spaces are frequently suggested disease mitigation strategies when traveling to known Chikungunya regions.^8^

The use of insect repellent as a means to protect against Chikungunya disease has been one area of focus of protective behavior education and research.^9,10^ However, avoiding exposure during peak daytime hours, wearing appropriate clothing (e.g. long sleeved shirts and pants), and using indoor spatial repellents (e.g., mosquito or spatial nets), are also effective protective behaviors.^11^ Wearing appropriate clothing while outdoors, such as long-sleeve shirts and pants, is a personal protective behavior frequently recommended to prevent transmission of vector-borne disease.^2,12-14^ One study on malaria found that wearing long sleeves and pants has a significant reduction in malarial incident 12-weeks post-exposure, more so, even, than repellants and insecticides.^15^ However, Omodior et al^16^ found that while nearly 75% of travelers to the Caribbean used insecticide and repellents during their outdoor time abroad, fewer than half that rate (31%) wore pants, long-sleeved shirts, boots, or hats when outdoors. As a result, given the lower rate of adoption of the latter personal protective behavior, investigation of the psychological constructs influencing appropriate clothing use is warranted. The use of indoor spatial repellents, such as a mosquito-repellent bed nets, represents another frequently-cited personal protective behavior against mosquito-borne disease.^12,17^ Window and door screens are also recommended mechanisms for reducing exposure while indoors.^13,14^ Among travelers to high-risk malaria destinations, approximately half report intention of using a mosquito net.^18^ However, adoption of this strategy may vary with perceived threat: In one study of travelers to countries with risk of malaria, dengue and Chikungunya, participants visiting high-risk malarial regions used bed nets 40%-60% of the time in contrast to only 5% of those in Chikungunya/dengue regions.^19^ This discrepancy in protective behaviors implicates a need for research into the factors that predict use of spatial netting against Chikungunya disease-carrying mosquitos.

The protectionmotivation theory (PMT) provides the theoretical framework for this investigation of individual adoption of two protective behaviors against the Chikungunya disease: Wearing appropriate protective clothing outdoors and using indoor spatial repellents.^20^ The PMT posits that four constructs of perceived vulnerability, perceived severity, perceived response efficacy, and perceived self-efficacy predict the likelihood of an individual adopting a protective behavior to avoid a health threat. Additionally, subjects’ knowledge of the threat is a requisite condition. In the context of this study, perceived vulnerability is the degree to which one believes there is a real possibility of contracting Chikungunya disease, whereas perceived severity is an assessment of its negative effects. Research indicates that higher perceived severity of a negative health outcome can induce behaviors that protect against disease or infection.^10,21^ Perceived response efficacy indicates the belief that one’s coping actions—wearing long clothes or a spatial net—is effective, whereas perceived self-efficacy reflects whether the individual believes they can perform the protective behavior. Floyd et al’s^22^ meta-analysis indicated that increases in these two coping variables facilitated adaptive behaviors. More recently, both perceived response efficacy and perceived self-efficacy were shown to be significant predictors of self-reported insect repellent use.^10^ This study will add to the body of literature by improving understanding of the theoretical constructs influencing adoption of two additional protective behaviors to prevent Chikungunya disease.

## Material and Methods

### Study design and participants

A cross-sectional study design using a survey instrument was conducted. The online, retrospective survey instrument was housed in Qualtrics^©^(http://www.qualtrics.com/) and study participants were recruited through Amazon^©^ Mechanical Turk (http://www.mturk.com/). Inclusion criteria consisted of age (over 18 years old), current residence in the United States, recent travel to one of 34 Caribbean destinations in the previous year for either leisure or vacation purposes, and engagement in at least one outdoor activity during that travel which might have resulted in exposure to mosquitos. A group of 653 adult U.S. international travelers met this initial criteria and responded to the survey Of these initial study participants, data for 184 individuals (28%) who completed all items on the questionnaire and who acknowledged on a dichotomous yes/no question that they had “heard about Chikungunya disease previously,” a requirement necessary to the PMT, were ultimately included. Power analysis for a logistic regression was conducted under the guidelines established in Allen and Le^23^ and using G*Power 3.1.7^24^ to determine a sufficient sample size using an alpha of 0.05, a power of 0.80, a medium effect size (odds ratio [OR] = 1.75) and two-tailed test. Based on these assumptions, the suggested sample size was below that of our final sample, indicating adequate size.

### Variables and measurements

Data collected from study participants included (a) sociodemographic information (age, gender, income, level of education, and race/ethnicity); (b) reported participation in outdoor activities while in the Caribbean (c) reported Chikungunya personal protective behaviors, i.e. (i) used bed nets, mosquito nets, insecticides or spatial repellents, and (ii) wore long-pants, long-sleeved shirts, boots, or hat; (d) responses that reflected the four theoretical constructs of the PMT, as well as respondents’, and (e) Chikungunya knowledge.

A detailed methodological explanation of the survey instrument and subsequent exploratory factor analysis has been published previously.^10^ In brief, the list of outdoor activities was developed from previously published scales.^25,26^ Single items reflecting a binary 0/1 scale were used to determine both self-reported use of appropriate clothing and indoor spatial repellents. Eleven items were used to assess the four theoretical constructs of the PMT, and internal reliability of each multi-item construct was evaluated using Cronbach α. Items were retained based on a Cronbach α threshold of ≥0.70.Four items measured perceived severity of Chikungunya and two items measured perceived vulnerability; both were calculated using an average of a 5-point Likert scale response (1 = strongly disagree, 5 = strongly agree). A single item was used to measure perceived response efficacy of appropriate clothing practices, while another was used to determine perceived response efficacy of indoor spatial repellent use. Two questions were used to determine perceived self-efficacy of indoor spatial repellent use, while perceived self-efficacy of appropriate clothing practices only necessitated one item. The same five-point Likert scale was utilized for these measures as well, and averages were calculated when constructs were measured by more than one item. Twelve item, adapted from the US Centers for Disease Control and Prevention and comprising a combination of true/false and multiple choice responses, were used to develop a Chikungunya knowledge score index.^27^

### Statistical analysis

Assumptions for logistic regression were evaluated following protocols outlined by Field.^28^ The assumption of linearity of the logic was not violated for either response variable; all continuous dependent variables were linearly related to the log of the outcome variable. Additionally, tests for multicollinearity indicated that several criteria for multicollinearity, including tolerance values, variance inflation factors, and variance proportions were within their target ranges. Absence of multicollinearity was further supported by results of bivariate correlations. Then, simple, unadjusted logistic regressions were performed to assess the impact of each of the five predictor variables—and the interactions between them—on the two protective behavior outcomes, measured on a dichotomous scale (0 = no, 1 = yes). Finally, a hierarchical multiple logistic regression was conducted to assess the combined ability of the four theoretical constructs and Chikungunya knowledge to explain additional variability for each outcome variable, beyond what was already explained by the sociodemographic variables. Data was analyzed using IBM^©^ SPSS version 25.0 (https://www.ibm.com/products/spss-statistics).

## Results

An exhaustive description of the study population has been published elsewhere.^10,16^ Of the 184 people included, nine percent of the sample (n = 17) reported income levels below $25 000, 29% (n = 54) reported income in the $25 001-$50 000 range, 31% (n = 57) reported income ranging from $50 001-$75,000, another 14% (n = 25) reported income of $75 001-$100 000, and 17% (n = 31) reported income above $100 000. There was nearly equal representation of gender identities, i.e., female (n = 91, 49.5%), male (n = 93, 50.5%). Age of participants ranged from 21 to 56 years old (mean = 32.8 years). Seven percent of the sample (n = 13) had at least a high school degree, 16% (n = 29) reported at least two years of college education, 66% (n =122) indicated attainment of a bachelor’s degree, and another 11% (n = 20) had completed a graduate program. After analyzing descriptive data for the study population, the relationships between Chikungunya knowledge and the theoretical constructs were investigated using the Pearson product moment correlation coefficient. Overall, results of the correlation analyses indicate several significant, but not strong, relationships (i.e., not greater than r = 0.70) between any two variables (Table 1).

### Wearing appropriate clothing

Simple, unadjusted logistic regression models predicting respondents’ reported practice of wearing appropriate clothing, are shown in Table 2. Results indicate several individual predictor variables that significantly predicted the participants’ self-report of wearing appropriate clothing. Then, an initial multiple logistic regression model was run to identify model outliers with standardized residuals ≥ 2.58. This resulted in the removal of n = 4 cases (2.2% of 184) for appropriate clothing use; these were cases in which predicted outcomes of the regression model significantly varied from the observed behavior. This listwise deletion increased classification accuracy from 74.5% to 80.0%.

Table 2 also reports the results of the hierarchical, multiple logistic regression predicting participants’ self-report of wearing appropriate clothing. Socio-demographic factors of age, gender, income, and education were entered in Step 1; together they explained between 14% (Cox & Snell R^2^ = 13.8) and 19% (Nagelkerke R^2^ = 19.1) of variability in respondents’ practices of wearing appropriate clothing, χ^2^ (9, 180) = 26.82, *P* = 0.001). In step 2 we added the theoretical constructs of perceived severity, perceived vulnerability, perceived response efficacy, perceived self-efficacy, and Chikungunya knowledge. This increased the total variance explained by the model as a whole to between 34% (Cox & Snell R^2^ = 33.9) and 47% (Nagelkerke R^2^ = 46.8), χ^2^ (14, 180) = 74.58, *P* = 0.001). In the final Step 3, four interaction terms were added (Chikungunya knowledge/perceived vulnerability, perceived response efficacy of appropriate clothing use/perceived self-efficacy of appropriate clothing use, Chikungunya knowledge/perceived Chikungunya severity, and perceived Chikungunya severity/perceived vulnerability); this resulted in a total variance explained by the model as a whole between 40% (Cox & Snell R^2^ = 39.7) and 55% (Nagelkerke R^2^ = 54.9), χ^2^ (18, 181) = 91.17, *P* < 0.001). After controlling for sociodemographic variables, the variables added in steps 2 and 3 explained between 26% (Cox & Snell R^2^ = 25.9%) and 36% (Nagelkerke R^2^= 35.8) of additional variability in respondents use of spatial repellents.

Among the theoretical constructs, perceived severity was significantly associated with the self-reported use of appropriate clothing. After adjusting for other variables, each unit increase in the average Likert-scaled score for perceived severity resulted in a slightly reduced odds of use of appropriate clothing (OR < 0.001, 95% CI = 0.00 – 0.020, *P* = 0.005). However, the inclusion of interaction terms indicated an opposite effect with regard to perceived severity. The interaction term between perceived Chikungunya severity and perceived vulnerability was associated with participants being more likely to wear appropriate clothing, after adjusting for other variables (AOR = 9.67, CI = 1.23-75.80, *P* = 0.031). Finally, the interaction term between Chikungunya knowledge and perceived severity was associated with participants being more likely to wear appropriate clothing (AOR = 1.95, CI = 1.18-3.25, *P* = 0.010) (Table 3).

### Indoor spatial repellent use

Simple, unadjusted logistic regressions were again conducted to predict the impact of each independent variable on respondents’ reported indoor spatial repellent use. Study results reveal that two terms significantly predicted indoor spatial repellent use (Table 3). Then, outliers were identified through a logistic regression that found 3 cases (1.6% of 184) in which predicted model outcomes differed significantly from the actual observed behavior (standardized residuals ≥ 2.58); listwise deletion of these outliers resulted in a change in classification accuracy from 78.3% to 79.6%.

Results of the hierarchical, multiple logistic regression predicting indoor spatial repellent use, inclusive of all independent variables, can be found in Table 3. Step 1 evaluated explanatory power of age, gender, income, and education; together they explained between 23% (Cox & Snell R^2^ = 23.4) and 31% (Nagelkerke R^2^ = 31.4) of variability in respondents’ indoor spatial repellent use, χ^2^ (9, 181) = 48.18, *P* < 0.001). After entry of the theoretical constructs of perceived severity, perceived vulnerability, perceived response efficacy, perceived self-efficacy, and Chikungunya knowledge, the total variance explained by the model was between 42% (Cox & Snell R^2^ = 41.6) and 56% (Nagelkerke R^2^ = 56.0) of variability in respondents’ indoor spatial repellent use, χ^2^ (14, 181) = 97.48, *P* < 0.001). Finally, in Step 3, four interaction terms were added (Chikungunya knowledge/perceived vulnerability, Perceived response efficacy of indoor spatial repellent use/perceived self-efficacy of indoor spatial repellent use, Chikungunya knowledge/perceived Chikungunya severity, and perceived Chikungunya severity/perceived vulnerability). This resulted in a total variance explained by the model as a whole between 46% (Cox & Snell R^2^ = 45.8) and 62% (Nagelkerke R^2^ = 61.6), χ^2^ (18, 181) = 110.94, *P* = 0.009). After controlling for sociodemographic variables, the variables added in steps 2 and 3 explained between 22% (Cox & Snell R^2^ = 22.4) and 30% (Nagelkerke R^2^= 30.2) of additional variability in respondents use of spatial repellents.

Among the theoretical constructs, Chikungunya knowledge was significantly associated with self-reported spatial repellent use. After adjusting for other variables, each unit increase in Chikungunya knowledge, measured on a 12-point scale, resulted a lower odd (0.02x) of indoor spatial repellent use (AOR = 0.02, CI = 0.00-0.31, *P* = 0.005). However, interaction between Chikungunya knowledge and other variables indicated an opposite effect. Each unit increase in the interaction term of Chikungunya knowledge and perceived vulnerability was associated with participants being more likely to engage in spatial repellent use, after adjusting for other variables (AOR = 1.88, CI = 1.18-3.02, *P* = 0.009). Finally, each unit increase associated with the interaction term of Chikungunya knowledge and perceived severity was associated with greater odds of using indoor spatial repellents (AOR = 1.55, CI = 1.05-2.29, *P* = 0.029).

## Discussion

This study explored the relationship between psychological variables and travelers’ adoption of specific protective health behaviors against Chikungunya disease among U.S. travelers to the Caribbean. This research is particularly relevant now, as the 30.6 and 29.9 million visitors that travelled to the Caribbean in 2017 and 2018 represented the 1^st^ and 2^nd^ highest number of travelers to the region in its history.^29^Industry officials projected that the region’s tourism sector would grow by 6%-7% in 2019, with cruise ship arrivals increasing by 4-5%.^29^Consequently, as Chikungunya disease and other vector-borne diseases spread globally due to climate change and increased global travel, understanding the psychological drivers of relevant protective behaviors is pivotal, especially given the lack of vaccinations or antiviral treatments against the disease.

Of the key constructs of the PMT, our results illustrate significant, negative relationships between (1) reported use of indoor spatial repellents and Chikungunya knowledge, (2) appropriate clothing use and perceived severity. However, several interaction terms were identified to be significant, positive predictors of the desired outcome behaviors. Notably, the interaction between perceived Chikungunya severity and Chikungunya knowledge predicted travelers’ practice of both (1) wearing appropriate clothing and (2) use of indoor spatial repellents. This finding suggests that knowledge alone in the presence of other variables did not significantly predict increased odds of adopting protective health behaviors, whereas its interaction with perceived Chikungunya severity did.

Additionally, the interaction between perceived Chikungunya severity and perceived vulnerability was found to be an additional predictor of travelers’ practice of wearing appropriate clothing; furthermore, the interaction of Chikungunya knowledge and perceived vulnerability was an additional predictor of indoor spatial repellent use. Notably, with both desired behaviors, increasing one construct alone was associated with lower odds of adopting the desired behavior (i.e., this was observed in Chikungunya knowledge in the case of spatial repellent use, and perceived Chikungunya severity in the case of wearing appropriate clothing). However, increasing two constructs—the interaction terms—was associated with a significant increase.

These results can be situated in a relatively inconclusive body of literature. One systematic review showed that risk perceptions, attitudes, and knowledge of chikungunya varied across populations and countries (the review, however, also largely represented Asian regions where the disease is endemic).^30^ However, among U.S. populations at risk to vector-borne or infectious diseases, significant relationships have been identified between perceived severity and indicators of heightened risk perception.^10^ Additionally, a broad review of protective behavior literature supports the general concept that higher appraisals of perceived risk influence health behavior.^31-33^ The results of our analysis aide in identifying strategies for health communication messaging and tourist education. While most literature studying the efficacy of educational interventions on disease prevention behaviors focus on endemic disease regions in the developing world, these strategies may also be transferable to international tourists from the developing world.^34,35^ Additionally, for both protective behaviors, given that the interaction between perceived Chikungunya severity and Chikungunya knowledge was significant, a two-pronged educational approach is recommended to (1) warn travelers of the disease’s severity as well as (2) increase their knowledge and understanding of it.

One limitation of this study is the use of self-reported data. It is possible that survey respondents, seeking to appear as effectively engaging in a desirable, protective behavior, over reported their adoption of indoor spatial repellent or appropriate clothing use.The data collection method (online) required that eligible study participants have internet access, which may have excluded portions of the targeted population. Whereas 74% of householders 15 years and over report internet access at home, fewer (a little over half, or 58.3%) of householders 65 years and older report household internet access.^36^This is particularly important as travel among the senior population (over 60 years of age) is anticipated to increase, and yet this population is also at an increased risk regarding the severity of Chikungunya disease.^37,38^

Despite these limitations, this research provides important information for tourism and public health officials to consider in order to reduce transmission of vector-borne diseases, like Chikungunya, among international travelers. For travelers whose home region is endemic to the vectors carrying disease (like *Aedes aegypti* and *Aedes albopictus* mosquitoes), this is particularly important to avoid new disease outbreaks. Increasing the degree to which travelers are knowledgeable about the disease and perceive it to be severe are important considerations for those designing education efforts. To this end, one direction for future research would to be increased understanding of why individuals choose not to engage in protective health behaviors while traveling, as the challenges in changing individual behaviors may vary between individuals who live in the region and others who are merely visiting. For instance, international travelers may be less willing to wear long-sleeved clothing while traveling to wear more fashionable or less confining, warm-weather clothing. Additionally, to identify nuances of specific locales and risks, future research might consider the role of the constructs of the PMT in the adoption of protection motivation behaviors within the context of other travel destinations and disease risks.

## Ethical approval

All research activities were approved by the University of Florida Institutional Review Board (IRB#: 2015-U-1040) with subject consent.

## Competing interests

The authors declare that they have no competing interests.

## Funding

This study received no funding or external support.

## Authors’ contributions

All authors were involved in the conceptualization of the study, revising the manuscript, or interpreting the results. Author KRA completed the analyses and drafted the first draft of the manuscript.

**Table 1:** Pearson product moment correlations between chikungunya knowledge, perceived severity, perceived vulnerability, response efficacy- & self-efficacy- for wearing appropriate clothing & use of indoor spatial repellents respectively

	**1**	**2**	**3**	**4**	**5**	**6**	**7**
1. Chikungunya knowledge	-	0.32 (*P* < 0.001)	0.22 (*P* = 0.002)	0.30 (*P* < 0.000)	-0.03 (*P* = 0.704)	0.26 (*P* < 0.000)	0.08 (*P* = 0.286)
2. Perceived severity		-	0.18 (*P* = 0.013)	0.20 (*P* = 0.004)	0.05 (*P* = 0.448)	0.073 (*P* = 0.310)	-0.05 (*P* = 0.496)
3. Perceived vulnerability			-	0.58 (*P* < 0.000)	0.43 (*P* < 0.000)	0.47 (*P* < 0.000)	0.16 (*P* = 0.029)
4. Perceived response efficacy of wearing appropriate clothing				-	0.54 (*P* < 0.000)		
5. Perceived self-efficacy for wearing appropriate clothing					-		
6. Perceived response efficacy of indoor protective measures			-			-	0.34 (*P* < 0.000)
7. Perceived self-efficacy for indoor protective measures							-

*Note.* Items 4 & 5 were specific to the first outcome variable (wearing appropriate clothing), while items 6 & 7 were specific to the second outcome variable (use of indoor protective measures. Pearson product moment correlation tests for these paired items were conducted only against items 1, 2, & 3. That is, items 4/5 were not tested for correlation against items 6/7 & vice-versa.

**Table 2:** Predictors of wearing appropriate clothing

**Predictor Values**	**Response Variable: Self-reported Wearing Appropriate Clothes**					
	**Unadjusted Models**			**Adjusted Model**		
	**UOR**	**95% CI**	***P*** **value**	**AOR**	**95% CI**	***P*** **value**
Perceived response efficacy	0.97	0.62-1.53	0.910	4.25	0.05-400.48	0.533
Perceived self-efficacy	0.71	0.50-1.01	0.056	7.35	0.11-516.38	0.358
Perceived severity	1.73	1.11-2.70	0.015*	<0.001	0.00-0.020	0.005**
Perceived vulnerability	0.75	0.48-1.15	0.187	0.01	0.00-6.14	0.168
Chikungunya knowledge	1.24	1.08-1.42	0.002***	1.78	0.19-16.64	0.613
Perceived response efficacy * perceived self-efficacy	0.97	0.92-1.03	0.345	0.56	0.19-1.67	0.297
Perceived Chikungunya knowledge * perceived vulnerability	1.03	0.96-1.10	0.444	9.67	1.23-75.80	0.031*
Chikungunya knowledge * perceived severity	1.06	1.03-1.08	0.000***	1.95	1.18-3.25	0.010**
Chikungunya knowledge * perceived vulnerability	1.02	0.10-1.05	0.102	0.54	0.27-1.09	0.087

*P value significance at *P* <0.05; **P value significance at *P* <0.01; ***P value significance at *P* <0.001. Abbreviation: UOR, unadjusted odds ratio; AOR, adjusted odds ratio. Note: Data reported under “unadjusted models” columns represents a model for each row. While not reported here, the hierarchical adjusted (multiple) logistic regression model also included socio-demographic factors (Step 1).

**Table 3:** Predictors of indoor spatial repellent use

**Predictor Values**	**Response Variable: Self-reported Use of Indoor Spatial Repellents**					
	**Unadjusted Models**			**Adjusted Model**		
	**UOR**	**95% CI**	***P*** **value**	**AOR**	**95% CI**	***P*** **value**
Perceived response efficacy	0.95	0.68-1.33	0.78	0.03	0.00-3.29	0.145
Perceived self-efficacy	1.95	1.40-2.71	0.00*	0.19	0.00-18.56	0.480
Perceived severity	1.31	0.87-1.96	0.197	11.29	0.03-4569.93	0.429
Perceived vulnerability	0.74	0.49-1.13	0.162	0.21	0.00-10.20	0.429
Chikungunya knowledge	1.01	0.91-1.13	0.796	0.02	0.00-0.31	0.005**
Perceived response efficacy * perceived self-efficacy	1.07	1.02-1.12	0.008**	2.20	0.73-6.66	0.163
Perceived Chikungunya severity * perceived vulnerability	1	0.94-1.07	0.974	0.23	0.04-1.24	0.087
Chikungunya knowledge * perceived severity	1.02	0.99-1.04	0.199	1.55	1.05-2.29	0.029*
Chikungunya knowledge * perceived vulnerability	0.1	0.98-1.02	0.894	1.88	1.18-3.02	0.009**

*P value significance at *P* <0.05; **P value significance at *P* <0.01. Abbreviation: UOR, unadjusted odds ratio; AOR, adjusted odds ratio. Note: Data reported under “unadjusted models” columns represents a model for each row. While not reported here, the hierarchical adjusted (multiple) logistic regression model also included socio-demographic factors (Step 1).
